# The emerging role of circular RNAs in common solid malignant tumors in children

**DOI:** 10.1186/s12935-021-01998-6

**Published:** 2021-06-11

**Authors:** Jiabin Yu, Li Yang, Hongting Lu

**Affiliations:** 1 Qingdao University, Qingdao, Shandong China; 2 Surgical Center of Women and Children’s Hospital, Qingdao University, No. 6, Tongfu Rd, Shibei District, Qingdao, 266011 Shandong China

**Keywords:** circRNA, Children, Malignant tumors, Solid tumors, Differential expression

## Abstract

Malignant tumors are one of the fatal diseases that threaten children’s physical and mental health and affect their development. Research has shown that the occurrence and development of malignant tumors are associated with the abnormal expression and regulation of genes. Circular RNAs (circRNAs) are noncoding RNAs that have a closed circular structure, with a relatively stable expression, and do not undergo exonuclease-mediated degradation readily. Recent studies have shown that circRNA plays an important role in the occurrence, metastasis, and invasion of solid malignant tumors (SMTs) in children. Thus, circRNA is being considered as a breakthrough in the treatment of SMTs in children. In this review, we describe the functions and mechanisms of circRNAs involved in SMTs in children oncogenesis, and summarize the roles of circRNAs in regulating cell proliferation, cell apoptotic death, the cell cycle, cell migrative and invasive ability, epithelial-mesenchymal transition (EMT), cancer stem cells and drug resistance in SMTs in children. In addition, we also discuss the role of circRNAs in the early diagnosis, pathological grading, targeted therapy, and prognosis evaluation of common SMTs in children. CircRNAs are likely to provide a novel direction in therapy in SMTs of children.

## Introduction

After accidental deaths, malignant tumors constitute one of the critical reasons for childhood deaths and are a serious threat to children’s health [1-2]. The solid malignant tumors (SMTs) in children mainly occur in the central, sympathetic nervous system, and mesenchymal tissues. Common ones include osteosarcoma, glioma, neuroblastoma, hepatoblastoma, infantile hemangioma, rhabdomyosarcoma, retinoblastoma, Wilms tumor, medulloblastoma and ependymoma, etc. The treatment methods include surgical resection, chemotherapy, and radiation therapy, but the treatment results are generally not favorable. Therefore, it is particularly important to explore new biomarkers and molecular mechanisms to treat SMTs in children. Several studies have shown that tumor development, cell invasion, proliferation, and apoptosis are closely related to the expression and regulation of noncoding RNAs. CircRNAs constitute a type of stable noncoding RNAs typically found in the cytoplasm of eukaryotic cells; however, a small part of intron-derived circRNA can be found in the nucleus. The traditional linear RNAs contain a 5′ end cap and a 3′ end poly(A) tail, but circRNAs have a closed-loop structure without 5′ and 3′ ends, have stable expression, and do not undergo endonuclease-mediated degradation readily [3]. Studies have shown that many circRNAs are differentially expressed in common SMTs in children and are involved in the occurrence and development of tumors. In this review, we will summarize the functions and mechanisms of circRNAs in SMTs in children oncogenesis and malignant progression.

### Overview of circRNA

CircRNA was first discovered by Sanger et al. [4] in RNA viruses in 1976. With the rapid development of high-throughput sequencing techniques, in 2012, Salzman et al. [5] were the first ones to detect more than 80 circRNAs in human leukocytes using RNA sequencing methods. Subsequently, Jeck et al. [6] discovered more than 25,000 circRNAs in human fibroblasts. Such a huge amount of data indicated that circRNAs were not accidental products during RNA splicing but were widely present, stable, and highly conserved in human cells. Most circRNAs are formed by reverse splicing of exons; however, a small part is formed by introns and intergenic regions. Based on their source, they are divided into exonic circRNAs (derived from exons) [7], intronic circRNAs (derived from introns) [8], exon-intron circRNAs (derived from exons and introns) [9], etc. Additionally, there exists a special type of circRNA, called tricRNA, which is formed by splicing from the precursor tRNA [10]. Studies have found that its expression is age-dependent and tissue-specific.

### Biological functions of circRNA

#### MiRNA sponge effect

CircRNAs absorb miRNA, like a sponge, via base complementation, and is referred to as the “miRNA sponge effect.” In 2013, two research teams reported that *CDR1*-derived circRNA (ciRS-7) could bind to and adsorb miR-7, reduce its activity, and indirectly upregulate the expression of miR-7-related target genes [11]. Zheng et al. found that circHIPK3 could directly bind to miR-124 and inhibit its activity [12]. In recent years, several studies have confirmed that the miRNA sponge effect of circRNA can be observed in various tissues and cell lines. Due to the stable structure of circRNAs, they exhibit better adsorption of miRNA than linear RNAs.

One of the essential functions of circRNAs includes transcriptional regulation. Li et al. [9] used cross-linked immunoprecipitation to study the effect of circRNA regulation on the transcription mechanism and identified the interaction between 111 circRNAs and RNA polymerase II in HeLa cells. CircEIF3J and CircPAIP2 knockout in HeLa and HEK293 cells, respectively, reduced the mRNA expression of the parent gene EIF3J and PAIP2. Also, the FMN gene is vital for limb development in mice. The exon circRNA is generated by reverse splicing during FMN gene transcription. Mice lacking this scissor acceptor are unable to detect exon circRNA expression. Although they have normal limb development, they will eventually develop the incomplete penetrance phenotype of renal hypoplasia [13]. CircRNAs reduce protein expression by blocking the translation start sites.

#### Interact with RNA binding proteins (RBP)

A study found that circRNAs can directly combine with RNAbinding proteins (RBP) to form an RNA-protein complex, which can regulate RBP or directly act on target genes via partial base pairing. They also interact with proteins indirectly via RNA-mediated and affect protein functions [14]. For example, circRNAs form a stable bond with AGO protein and RNA polymerase II and enhance gene transcription by increasing its transcription function. Exon circRNAs also serve as a “scaffold” for RNA-binding proteins by binding to multiple proteins to strengthen the stability of their interactions. Studies have also suggested that circRNAs can be used as a targeting element while binding to RBP and complementary RNA or DNA sequences [7]. The splicing factor MBL is a type of RBP. In humans and Drosophila, it can combine with the second exon of its parent gene to promote its cyclization to form circMbl, which can combine with MBL, reduce the effective concentration of MBL, ultimately reducing the synthesis of circMbl [15].

#### Participate in protein translation

Some circRNAs can encode proteins. The hepatitis delta factor, present in mammalian cells, is a protein encoded by circRNA [16]. Studies have shown that circRNAs in U20S, a human osteosarcoma cell line, exhibit low-efficiency protein translation [17]. Legnini reported that circ-ZNF609 could bind to polyribosomes and participate in the transcription and translation of proteins through 5′ cap-independent and splicing-independent forms [18].

## CircRNA and common SMTs in children

In the following sections, we summarize the current research on the specific role and mechanism of circular RNA in the progression of SMTs in children (Fig, 1, Table 1).

**Fig. 1:**
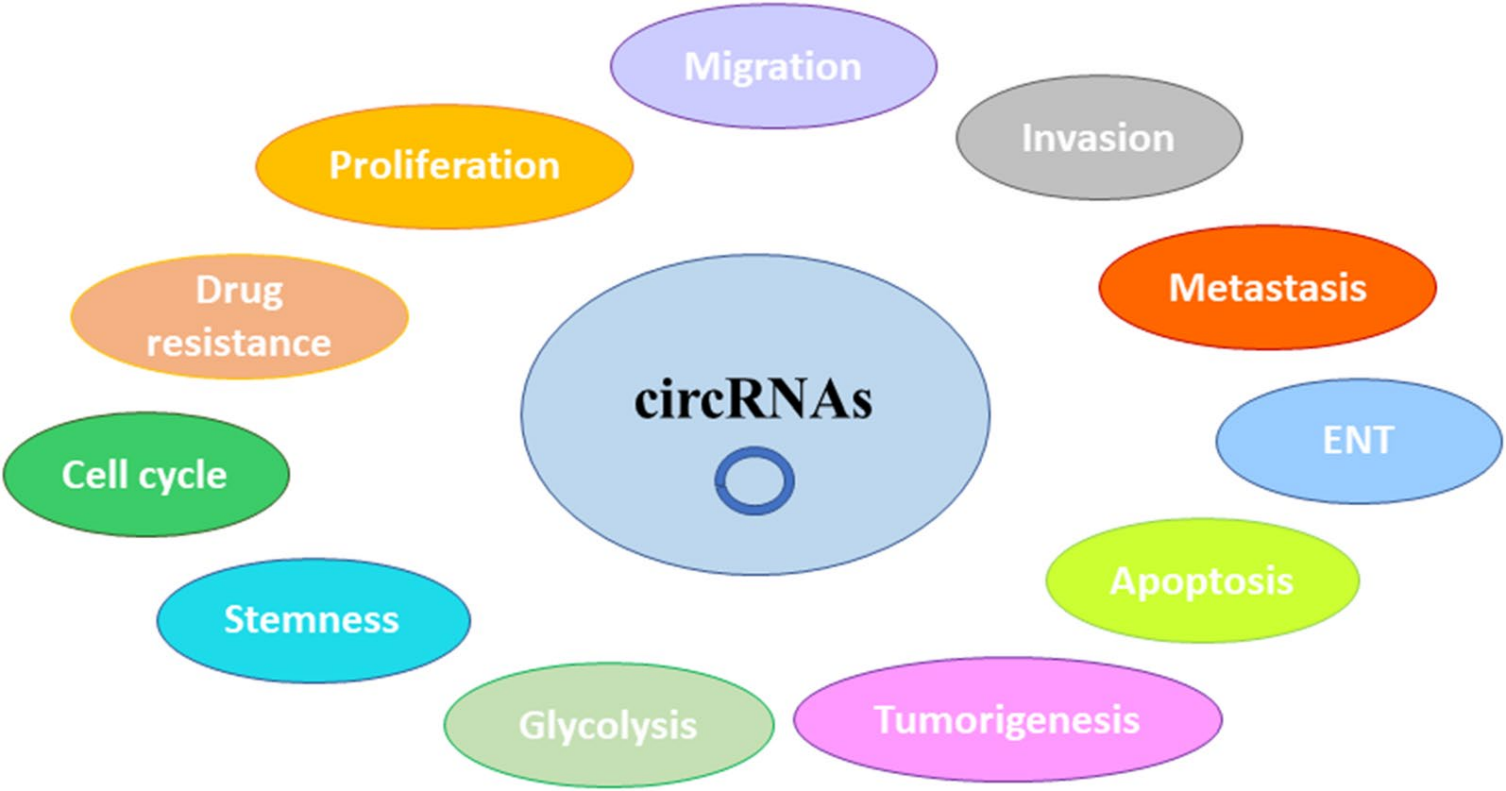
The role of circRNAs in regulating cellular processes. CircRNAs play a critical role in the regulation of cell proliferation, cell migration and invasion, epithelial-mesenchymal transition (EMT), cell apoptotic death, tumorigenesis, glycolysis, stemness, cell cycle, and drug resistance in SMTs in children

**Table 1:** Role of circRNAs in solid malignant tumors (SMTs) in children

SMTs in children	CircRNA	Expression	Target gene/protein	Biological function	Ref
Osteosarcoma	Circ_0001105	Down	MiR-766/YTHDF2	Inhibit proliferation, migration, and invasion	[19]
	CircTADA2A	Up	MiR-203a-3p/CREB3	Promote progression and metastasis	[20]
	CircMYO10	Up	MiR-370-3p/RUVBL1	Promote proliferation and EMT	[21]
	Circ-0008717	Up	MiR-203	Promote proliferation, migration, and invasion	[22]
	CircFAT1	Up	MiR-375/YAP1	Promote migration, invasion, and tumorigenesis	[23]
	Hsa_circ_0000282	Up	miR-192/XIAP	Promote proliferation	[24]
	circ_0001649	Down	MiR-338-5p/miR-647/miR-942	Inhibit proliferation, migration; induce apoptosis	[25]
Glioma	Circ_002136	Up	MiR-138-5p/SOX13	Promote angiogenesis	[26]
	CircHIPK3	Up	MiR-124-3p	Promote proliferation, migration, and invasion; inhibit apoptosis	[27]
			MiR-654/IGF2BP3	Promote proliferation, migration, and invasion	[28]
Neuroblastoma	CircAGO2	Up		Promote proliferation, migration, and invasion	[29]
	CircCUX1	Up	EWSR1/MAZ	Induce the aerobic glycolysis	[30]
		Up	miR-16-5p/DMRT2	Promote proliferation, migration, invasion, and glycolysis	[31]
Hepatoblastoma	Circ_0015756	Up	MiR-1250-3p	Promote proliferation and invasion	[32]
	CircHMGCH1	Up	MiR-503-5p/IGF2	Promote proliferation	[33]
	CircSTAT3	Up	MiR-29a/b/c-3p	Promote proliferation, invasion, migration, stemness	[34]
Infantile hemangioma	Hsa_circ_100933	Up			[35]
	Hsa_circ_100709	Up			[35]
	Hsa_circ_104310	Down			[35]
	CircAP2A2	Up	MiR-382-5p/VEGFA	Promote proliferation, migration, and invasion	[37]
Rhabdomyosarcoma	Circ-ZNF609	Up		Regulate G1-S progression	[38]
Retinoblastoma	Circ-0075804	Up	E2F3	Promote proliferation	[39]
	CircVAPA	Up	miR-615-3p/SMARCE1	Promote proliferation, migration, and invasion	[40]
	Hsa_circ_0001649	Up	AKT	Promote proliferation, migration, and invasion; inhibit apoptosis	[41]
Wilms’ tumor	Circ_0017247	Up	MiR-145	Induce metastasis	[42]
Medulloblastoma	Circ-SKA3	Up		Promote the proliferation, migration and invasion	[43]
	Circ-DTL	Up		Promote the proliferation, migration and invasion	[43]

### Osteosarcoma

Osteosarcoma (OS) is a type of malignant bone tumor commonly found in adolescents or children. It constitutes approximately 5% of pediatric tumors. Yang et al. [19] tested the cancerous tissues, paracancerous tissues, and cell lines (U2OS, MG63) of 30 OS patients using qRT-PCR and found a significantly lower expression of circ_0001105 and YTHDF2 in OS tissues and cell lines compared with paracancerous tissues, while miR-766 levels were substantially elevated than the adjacent tissue. Also, the expression of circ_0001105 in patients with advanced cancer, metastatic tumors, and recurrent tumors was considerably higher, which indicated that downregulated expression of circ_0001105 was related to the prognosis and survival of patients. Regarding functional tests, ectopic overexpression of circ_0001105 or YTHDF2 significantly inhibited the proliferation, migration, and invasion of OS cells via the regulation of miR-766. In vivo experiments also confirmed that overexpression of circ_0001105 could inhibit tumor growth. Similarly, Wu et al. [20] showed substantially increased expression of circTAD2A in OS tissues and cell lines. Occurrence and metastasis suggested that circTAD2A-miR-203a-3p/CREB3 could be used as a new target for the treatment of OS. CircMYO10 activated H4K16Ac in the promoter region of the β-protein/LEF1 target gene by regulating the miR370-3p/RUVBL1 axis, thereby activating the Wnt/β-protein signal transduction pathway [21]. In OS cells, circRNA-0008717 could directly bind to miR-203 and promote OS progression via sponge effect and upregulation of BMI-1 expression [22]. CircFAT1 promoted YAP1 expression in OS cells by sponging miR-375 [23]. Hsa_circ_0000282 acted as a competitive endogenous RNA and promoted OS cell proliferation by regulating miR-192/XIAP axis [24]. circ_0001649 can inhibit the viability and survival of U2OS and HOS cells by sponging miR-338-5p, miR-647 and miR-942, induce apoptosis and activate the STAT signaling pathway [25].

### Glioma

Glioma is the most common primary central nervous system tumor, accounting for about half of all intracranial primary tumors. Most children get sick around 10 years of age. CircRNAs are known to promote angiogenesis in gliomas [26]. Studies have observed elevated levels of circ_002136 in gliomas, and its knockdown could suppress angiogenesis in gliomas. MiR-138-5p is known to target and regulate circ_002136 and reverse circ_002136-mediated angiogenesis of gliomas. Bioinformatic studies by He et al. identified the target gene SOX13 of miR-138-5p involved in angiogenesis. Functional analysis found that miR-138-5p inhibited SOX13-mediated angiogenesis and SPON2 expression. SOX13 promoted FUS transcription to form a feedback loop that regulated glioma angiogenesis. FUS, as an RBP, promoted the generation of circRNA to regulate angiogenesis in gliomas. Also, studies by He et al. demonstrated the presence of a feedback loop in the angiogenesis of glioma, namely FUS (RBP), circ_002136 (CircRNA), miR-138-5p (MiRNA), SOX13 (transcription factor), and SPON2 Target genes, a multi-level molecular regulatory network. Other studies have shown that circRNA expression affects the cell cycle in glioma cells [27]. Targeted siRNAs-mediated knockdown of circHIPK3 has been shown to substantially suppress cell proliferation and invasion as well as arrested G0/G1 cell cycle while inducing apoptosis. After further experimentation, Jin et al. [28] speculated that circHIPK3 affected cell proliferation, invasion, and metastasis of glioma cells through the circHIPK3/miR-654/IGF2BP3 regulatory network. The experimental evidence suggested that circHIPK3 was a potential therapeutic target for the treatment of gliomas.

### Neuroblastoma

Neuroblastoma (NB), a common extracranial tumor, is usually found in infants and young children. NB is a neuroendocrine tumor, which can originate from any nerve ridge of the sympathetic nervous system. Chen et al. [29] found an upregulated expression of circAGO2 in NB tissues and cell lines. They speculated a relationship between circAGO2 and cancer growth, invasion, and metastasis and were able to experimentally prove the interaction between circAGO2 and HuR in cancer cells, followed by the activation of the latter. By promoting the HuR inhibitory function of the AGO2-miRNA complex, AGO2-miRNA-mediated gene silencing was suppressed, leading to increased target gene expression; thereby, promoting the occurrence and invasion of tumors. Thus, circAGO2 could help with the development of strategies to diagnose NB. They further found that circAGO2 knockout increased the survival probability of nude mice. This preliminary evidence suggested that circRNAs played an important role in the prognosis of NB. The experimental study by Li et al. [30] found that the intron-containing circular RNA (circ-CUX1) produced by the oncogene *CUX1* was vital for the occurrence and invasion of NB. QRT-PCR analysis detected the upregulation of circCUX1 in NB tissues and cell lines. The upregulated circ-CUX1 bound to EWSR1 to promote its interaction with MYC-related zinc finger proteins to induce the aerobic glycolysis in NB. Subsequent functional studies found that circCUX1 had a carcinogenic role in the progression of NB. Transfected cell lines with stable circCUX1 expression exhibited enhanced glycolysis and reduced oxygen consumption. After stable transfection of circCUX1 in IMR32 cells, a substantial increase in growth, tumor weight, Ki-67 proliferation index, CD31-positive microvessels, glucose uptake, lactate production, and ATP levels in nude mice was observed. Finally, they discovered that circ-CUX1 targeted miR-16-5p/DMRT2 signaling cascade to regulate the proliferation, migration, invasion, and glycolysis of NB cells [31].

### Hepatoblastoma

Hepatoblastoma (HB), a malignant embryonic tumor, is a common liver tumor in children. Liu’s study used gene sequencing to detect the differential expression of circRNA in the HB tissue and adjacent tissue [32]. They found a substantially upregulated expression of circ_0015756 in HB tissues. Functional studies found that circ_0015756 acted as a miR-1250-3p sponge to regulate HB cell function. Circ_0015756 knockdown reduced HB cell viability, proliferation, and invasion in vitro. They also found considerably elevated circ_0015756 expression in the peripheral blood of patients with HB. Similarly, Zhen et al. [33] also found a substantially heightened circHMGCS1 expression in HB tissues of 64 hepatectomy patients and normal liver tissues, as well as observed that circHMGCS1 was regulated by the “sponge” miR-503-5p, the expression of IGF2 and IGF1R, and affected the downstream PI3K-Akt signaling pathway, regulated HB cell proliferation. This suggested that circHMGCS1 could be a promising therapeutic target and prognostic marker for HB. Liu et al. [34] found that circ-STAT3 acted as an miR-29a/b/c-3p sponge to elevate STAT3 and Gli2 expression with Gli2 as the transcription factor for circ-STAT3. Circ-STAT3 facilitated cell proliferation, invasion, migration, stemness, and tumor growth in HB by upregulating STAT3 and Gli2.

### Infantile hemangioma

Infantile hemangioma (IH) is a congenital benign tumor or vascular malformation induced by the proliferation of hemangioblasts during embryo formation and is common in skin and soft tissues. It is commonly detected either at the time of birth or shortly after birth. Fu et al. [35] were the first ones to analyze circRNAs in IH, identified the circRNAs that were differentially expressed, and identified 234 upregulated, and 374 downregulated circRNAs. Among them, hsa_circ_100933 and hsa_circ_100709 were upregulated, and hsa_circ_104310 was downregulated. Similarly, Li et al. [36] conducted a study on the proliferating capillary hemangioma and matching normal skin tissues, obtained from 3 children, and found 249 differentially expressed circRNAs (DE-circRNAs) via qRT-PCR. There were 124 upregulated and 125 downregulated circRNAs in the IH tissue. The expressions of hsa_cicRNA_001885 and hsa_cicRNA_006612 in hemangioma tissue are 12.33 times and 7.13 times higher than that of normal skin, respectively. They also analyzed the source genes of DE-circRNAs via GO and KEGG pathway analysis. GO analysis showed that genes derived from DE-circRNAs are mainly involved in the generation of cellular components, protein binding, and chemical components in cells. KEGG pathway analysis showed that the genes of origin of DE-circRNAs were mainly related to information exchange between cells. CircAP2A2 was highly expressed in IH [35]. Downregulated expression of circAP2A2 could substantially inhibit growth, proliferation, invasion, and migration of IH cells. Yuan et al. [37] performed bioinformatics analysis and functional studies and showed that circAP2A2 regulated VEGFA expression through sponge miR-382-5p, promoting the development of hemangioma. Therefore, circAP2A2/miR-382-5p/VEGFA axis might provide new ideas for the treatment of IH.

### Rhabdomyosarcoma

Rhabdomyosarcoma (RMS) is the most common type of soft tissue sarcoma in children. It is a malignant tumor that originates from striated muscle cells or mesenchymal cells that differentiate into striated muscle cells. Rossi et al. [38] studied the expression and function of circ-ZNF609 in children with RMS to understand the role of circ-ZNF609 in cell cycle regulation. They found that circ-ZNF609 was mainly upregulated in embryonic and alveolar RMS biopsies. Also, circ-ZNF609 knockout in embryonic RMS-derived cell lines resulted in a substantial decrease in p-Akt protein levels, vital for the cell cycle. Thus, it was hypothesized that circ-ZNF609 acted as a new regulator of cell proliferation pathways by resisting p-Akt proteasome-dependent degradation. Also, Rossi et al. found that circ-ZNF609 downregulated cell cycle-related genes and upregulated innate immune genes via RNA sequence analysis of human primary myoblasts lacking circ-ZNF609, indicating a close relationship between circ-ZNF609 and RMS cell proliferation.

### Retinoblastoma

Retinoblastoma (RB), a malignant tumor derived from photoreceptor precursor cells, is usually found in children under 3 years old, with family genetic predisposition. Circ-0075804 promotes RB cell proliferation by binding to heterogeneous ribonucleoprotein K (HNRNPK), thereby increasing the stability of E2F3 mRNA, which indicates that circ-0075804 may become a therapeutic target for RB patients [39]. Xu found that circVAPA was upregulated in human RB specimens and RB cell lines through qRT-PCR, and was associated with the poor prognosis of RB patients. Knockout of circVAPA can inhibit the malignant phenotype of RB. After studying the mechanism, he found that miR-615-3p can reverse the RB cell effect induced by circVAPA, and circVAPA positively regulates the downstream oncogene SMARCE1 through miR-615-3p. In addition, in vivo nude mice tumor formation experiments confirmed this finding [40]. Xing et al. found that the down-regulation of the circular RNA hsa_circ_0001649 indicates that the prognosis of retinoblastoma is poor, and it regulates cell proliferation and apoptosis through the AKT/mTOR signaling pathway [41]. Which indicates that strengthening the effect of hsa_circ_0001649 may be a potential treatment strategy for RB.

### Other SMTs in children

Children’s SMTsare not only limited to the above-mentioned types, but also Wilms’ tumor, medulloblastoma, ependymoma, etc. Studies have found that circ_0017247 induces Wilms’ tumor metastasis through targeting miR-145 [42]. circ-SKA3 and circ-DTL promote the proliferation, migration and invasion of medulloblastoma cells by regulating the expression of host genes. This shows that circ-SKA3 and circ-DTL have key carcinogenic effects in the occurrence and development of medulloblastoma [43]. Ahmadov et al. found that ependymoma specifically upregulated circRNA derived from RMST, LRBA, WDR78, DRC1 and BBS9 genes through Next Generation Sequencing, but their results still need experiments to prove [44].

## Conclusion

With the advancements in high-throughput sequencing techniques, extensive studies are being conducted to understand the role of circRNAs in various diseases. Due to the specific structure of circRNA, it is highly conserved and abundantly present in cells. Currently, it can be regarded as a research hotspot. Particularly, the relationship between circRNA and tumors cannot be ignored. Several studies have shown that circRNAs play a vital role in tumor occurrence, proliferation, metastasis, invasion, EMT, apoptosis, and cell cycle. The dysregulated circRNAs between SMTs in children and normal cells/tissues may participate in regulating the inhibition and promotion of these SMTs in children, suggesting that circRNAs could serve as therapeutic targets and biomarkers for clinical diagnosis. However, the above assumptions continue to present substantial difficulties and enormous challenges in clinical practice, and more studies on the significance, efficiency, security, and reliability of these approaches are needed. Thus, we expect that this review will increase the comprehension of the principal functions of many circRNAs and their multiple regulatory hubs in the progression of SMTs in children. We are optimistic that the use of sequencing technologies will elucidate roles of circRNAs implicated in SMTs in children tumorigenesis may eventually accelerate the clinical application of circRNAs for use in diagnosis, treatment, and prognosis evaluation.

## Data Availability

Not applicable.
